# Comparison of D-dimer with CRP and ESR for diagnosis of periprosthetic joint infection

**DOI:** 10.1186/s13018-019-1282-y

**Published:** 2019-07-29

**Authors:** Longjiang Xiong, Siyun Li, Min Dai

**Affiliations:** 1 Nanchang University, Nanchang, 330003 China; 2 Department of Orthopedics, Jiangxi Province Hospital of Integrated Chinese and Western Medicine, Nanchang, 330003 China; 3 Department of Orthopedics, The First Affiliated Hospital of Nanchang University, NO.17, Yongwai Street, Nanchang, 330006 China

**Keywords:** Periprosthetic joint infection, Biomarkers, D-dimer, Erythrocyte sedimentation rate, C-reactive protein

## Abstract

**Background:**

Despite the availability of several biomarkers, the diagnosis of periprosthetic joint infection (PJI) continues to be challenging. Serum D-dimer assessment is a widely available test that detects fibrinolytic activities and has been reported as an inflammatory biomarker. However, quite a few articles have reported the diagnostic efficiency of D-dimer for PJI.

**Methods:**

This prospective study enrolled patients who had undergone total joint arthroplasty, were suspected of PJI, and also prepared for revision arthroplasty. PJI was defined using the Musculoskeletal Infection Society criteria. In all patients, serum D-dimer level, erythrocyte sedimentation rate (ESR), and C-reactive protein (CRP) level were measured preoperatively. We then compared the diagnostic efficiency of these three biomarkers.

**Results:**

The median D-dimer level was significantly higher (*p* < 0.001) for the patients with PJI than for the patients with aseptic failure. With a sensitivity of 80.77% (95% CI, 65.62 to 95.92%) and a specificity of 79.63% (95% CI, 68.89 to 90.37%), the diagnostic efficiency of D-dimer did not outperform serum CRP (with a sensitivity of 84.61% and specificity of 64.81%) and ESR (with a sensitivity of 73.08% and specificity of 90.47%).

**Conclusions:**

Serum D-dimer as a marker for the diagnosis of PJI still requires more large-scale and detailed clinical trials.

## Background

Periprosthetic joint infection (PJI) after total hip arthroplasty (THA) or total knee arthroplasty (TKA) is one of the most dreadful complications and it has extremely negative effects on the physical, emotional, social, and economic aspects of a patient’s life [1, 2]. Currently, an absolute test for the diagnosis of PJI does not exist, compelling clinicians to rely on a combination of synovial fluid tests and serological markers [3]. Due to the lack of an absolute test, the Musculoskeletal Infection Society (MSIS) introduced a set of diagnostic criteria for PJI.

The MSIS guidelines include two major and six minor diagnostic criteria, with the latter involving measurements of serum C-reactive protein (CRP) level, erythrocyte sedimentation rate (ESR), synovial fluid white blood cell (WBC) count, and neutrophil differential, culture, and leukocyte esterase testing. Although numerous serological markers for PJI have been evaluated in the past, including interleukin 6 (IL-6), erythrocyte sedimentation rate (ESR) and C-reactive protein (CRP) have been generally used as a screening test for infection because of their simplicity and cost-effectiveness [4, 5]. However, they have low sensitivity and specificity and these may increase under several conditions in addition to infection [6, 7]. A recently introduced test of synovial fluid biomarker, namely alpha-defensin, has been reported to diagnose PJI with high sensitivity and specificity, but its optimal threshold for the diagnosis of PJI remains unknown and also it is expensive [8]. The combined measurement of synovial fluid alpha-defensin and CRP for the diagnosis of PJI demonstrated a sensitivity of 97% and a specificity of 100% [9]. However, it frequently occurs when either an inadequate amount of fluid is available to perform all tests or, worse, no fluid is retrieved from the joint [10]. Obtaining synovial fluid is invasive and painful to patients. In addition, there is a theoretical, yet real, concern for the introduction of infection into the joint and, in difficult aspirations, especially in the hip, contamination of the aspirated fluid may occur, leading to false-positive results [11].

The issues mentioned above highlight the requirement for a reliable serological test that can help to diagnose PJI. D-dimers are fibrin degradation products formed as a result of fibrin clot dissolution by plasmin. D-dimer assessment was traditionally used as a screening test for detecting venous thromboembolism (VTE) but was largely abandoned because of its poor accuracy [12]. More recently, assessment of serum D-dimer has gained attention for its role in predicting poor outcome in sepsis and bacteremia [13].

Therefore, in this study, we were interested in whether patients with PJI have a higher level of circulating D-dimer and whether D-dimer could be indicative of infection in patients suspected of PJI.

## Methods and materials

Ethical approval for this prospective study was obtained from the Ethics Committee of our hospital. From April 2017 to August 2018, patients who had undergone primary total joint arthroplasty (TJA) and were then suspected of PJI were included in our study. Excluded were patients who have taken preoperative pharmacologic deep vein thrombosis (DVT) prophylaxis or suspicious DVT, with inflammatory arthritis such as rheumatoid arthritis, hematoma, a history of recent trauma or dislocation (within 2 weeks), visible ecchymosis, or a history of hypercoagulation disorder. Patients were divided into PJI group and non-PJI group based on the MSIS criteria. PJI is present when one of the major criteria exists or four out of six minor criteria exist: (1) elevated serum CRP and ESR (CRP > 10mg/d, ESR > 30 mm/h), (2) elevated synovial fluid white blood cell (WBC) count (> 3000 cells/μL), (3) elevated synovial fluid polymorphonuclear neutrophil percentage (PMN > 65%), (4) presence of purulence in the affected joint, (5) isolation of a microorganism in one periprosthetic tissue or fluid culture, and (6) > 5 neutrophils per high-powered field in 5 high-power fields observed from histologic analysis of periprosthetic tissue at × 400 magnification.

We recorded patients’ sex and age and the involved joint. Moreover, concurrent antibiotic treatment (excluding a single dose of prophylactic perioperative antibiotic) and isolated organisms were noted for all of the patients. Venous blood samples were obtained preoperatively on the day before surgery, which were then analyzed for serum D-dimer, ESR, and CRP. The D-dimer level in was assessed using an immunoturbidimetric assay on a STA-R analyzer (Diagnostica Stago).

### Statistical analysis

Descriptive statistics were used to report all of the laboratory values. Comparison of ESR, CRP, and D-dimer between preoperative and postoperative values was performed using a paired *t* test. The optimal threshold for D-dimer as diagnostic of PJI was determined by the Youden *J* statistic (*J* = sensitivity + specificity − 1) based on its correspondence with the diagnosis of PJI. The sensitivity and specificity of the diagnostic tests were calculated along with their 95% confidence intervals (CIs). All statistical analyses were performed using GraphPad Prism (version 7.0a; GraphPad Software).

## Results

Based on the MSIS criteria, 26 patients (14 knee replacement and 12 hip replacement) were diagnosed as the PJI group, while the other 54 patients (33 knee replacement and 21 hip replacement) were defined as the non-PJI group. In the PJI group, 7 were males and the other 19 were females, with age ranging from 41 to 78 (65.42 ± 10.8) and body mass index (BMI) of 20.20~34.74 kg/m^2^ (25.07 ± 5.32 kg/m^2^). Among the 54 uninfected patients, there were 25 males and 29 females, with age ranging from 42 to 76 (59.76 + 12.53) and BMI of 19.06~32.68 kg/m^2^ (22.87 ± 3.77 kg/m^2^). There was no statistical difference between these two groups in terms of age, gender, and BMI (*p* > 0.05).

**Fig. 1:**
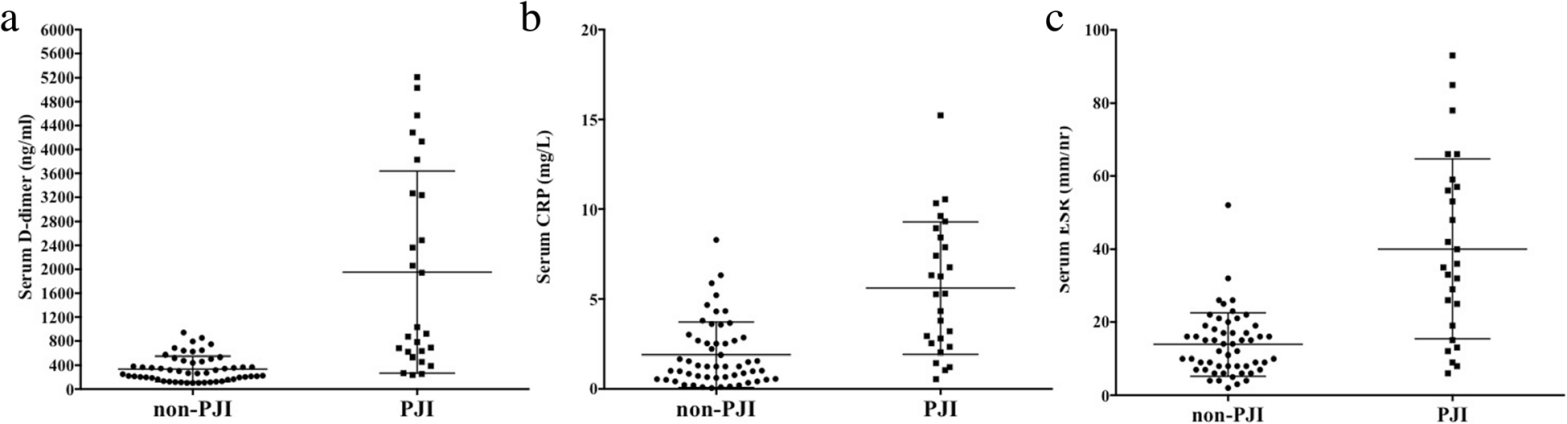
Serum D-dimer (**a**), CRP (**b**), and ESR (**c**) significantly increased in PJI group, compared with the non-PJI group (*p* < 0.001)

Before surgery, serum D-dimer level was significantly higher in patients with PJI; the mean D-dimer level was 1953.35 ng/mL (238 to 5211 ng/mL) in the PJI group, compared with 336.50 ng/mL (103 to 942 ng/mL) in the non-PJI group (*p* < 0.001) (Fig. 1a). The median ESR and CRP values were also significantly higher among the patients with PJI. The median ESR was 40.04 mm/h (6 to 93 mm/h) in the PJI group, compared with 13.87 mm/h (2 to 52 mm/h) in the non-PJI group (*p* < 0.001) (Fig. 1b). The mean CRP was 5.61 mg/L (0.50 to 15.23 mg/L) in the PJI group, compared with 1.9 mg/L (0.55 to 8.30 mg/L) in the non-PJI group (*p* < 0.001) (Fig. 1c).

Compared with the MSIS criteria, the serum D-dimer test demonstrated a sensitivity of 80.77% (95% CI, 65.62 to 95.92%) and a specificity of 79.63% (95% CI, 68.89 to 90.37%), for diagnosing PJI. Assessments of serum CRP level and ESR demonstrated a sensitivity of 84.61% (95% CI, 70.74 to 98.48%) and 73.08% (95% CI, 56.03 to 90.13%) and a specificity of 64.81% (95% CI, 52.41 to 74.81%) and 90.47% (95% CI, 82.74 to 98.47%), respectively (Table 1). With Youden’s index, 756 ng/ml was determined as the optimal threshold value for serum D-dimer for the diagnosis of PJI.

**Table 1:** Performance of serum tests for diagnosing PJI

	D-dimer	CRP	ESR
True positive	21	22	19
False positive	11	19	5
False negative	5	4	7
True negative	43	35	49
Sensitivity (95%CI)	80.77% (65.62–95.92%)	84.61% (70.74–98.48%)	73.08% (56.03–90.13%)
Specificity (95%CI)	79.63% (68.89 90.37%)	64.81% (52.41–74.81%)	90.47% (82.74–98.47%)
AUC (95%CI)	0.890 (0.814–0.966)	0.831 (0.737–0.926)	0.838 (0.732–0.944)

*PPV* positive predictive value, *NPV* negative predictive value, *+LR* positive likelihood ratio, *−LR* negative likelihood ratio

## Discussion

Serum D-dimer is a fibrin degradation product released into the blood following the fibrin clot breakdown by plasmin and has been used for screening patients for venous thromboembolism (VTE) [14]. In recent years, evidence has emerged to suggest that D-dimer levels are likely to rise in the setting of systemic inflammation and infection, especially in the joint [13, 15, 16]. A persistent inflammatory response could contribute to a hypercoagulable state, possibly via cytokine-induced activation of the endothelium or by induction of monocytes to express tissue factor [17]. D-dimer and other fibrin degradation products may influence inflammatory and acute-phase responses by promoting neutrophil and monocyte activation [18]. In addition, it has been demonstrated that D-dimer levels increased in patients with rheumatoid arthritis (RA) and it might be considered as a biomarker for diagnosis of RA [19, 20]. Activation of the coagulation and fibrinolytic cascades in the joint and in the circulation could be observed in both inflammatory and degenerative joint diseases [21]. So et al. [22] demonstrated that within the joint, inflammatory mechanisms leading to the activation of the coagulation pathway and the increased amounts of thrombin-activated fibrinolysis inhibitor (TAFI) in RA might explain why fibrin formation is so prominent when compared with other joint diseases. The increased fibrinolytic activity and generation of byproducts such as D-dimer are considered to localize in the infecting organisms or inflammatory cells, thus preventing them from triggering systemic damage [23, 24]. The byproduct of this fibrinolytic activity also “leaks” into the circulation and can thus be measured [25]. Recently, D-dimer has shown its promise as a biomarker for the diagnosis of PJI, as well as timing of reimplantation [26].

From our study, we compared the diagnostic efficiency of serum D-dimer with CRP and ESR for PJI diagnosis. The sensitivity and specificity of D-dimer fall between the sensitivity and specificity of serum CRP and ESR through the evaluation of 80 patients suspected of PJI. In a prospective study conducted by Shahi et al. [26], they reported that serum D-dimer outperformed both ESR and serum CRP, with a sensitivity of 89.5% and specificity of 92.8%. They also believed that “elevated” D-dimer at the time of reimplantation could predict the infection, which caused subsequent failure. While for those false-positive results, they considered it as the infection caused by slow-growing organisms that did not elicit physiological inflammation and failed to meet the MSIS criteria for PJI. In another study conducted by Lee et al. [27], D-dimer was proved as effective in early detection of PJI if combined with levels of ESR and CRP. However, this study used the data of primary surgery and did not further compare the difference between postoperative infections with those who were not infected.

There are several limitations in our current study. Firstly, the number of patients included in this study was still limited due to the different hospital stays of patients. For some patients who were eligible for other inclusion criteria, we could not obtain the serum results of CRP, ESR, and D-dimer on the third or fifth day after surgery. Secondly, more postoperative data should be acquired to further validate the rise and fall tendency of these three tests.

## Conclusions

In conclusion, we found that serum D-dimer assessment might be comparable with serum CRP and ESR for diagnosis of PJI. In addition, the rise and fall of serum D-dimer is more rapid than serum CRP and ESR postoperatively. This test might be used effectively in diagnosing early postoperative infection and this progress might be considered as a baseline for early diagnosis of infection. Further validation work is still required to reproduce these findings and confirm the relative test performance of D-dimer versus other more established serum markers.

## Data Availability

The datasets used and/or analyzed during the current study are available from the corresponding author on reasonable request.

## Abbreviations

C-CRP: Reactive protein
CIs: Confidence intervals
ESR: Erythrocyte sedimentation rate
MSIS: Musculoskeletal Infection Society
PJI: Periprosthetic joint infection
TAFI: Thrombin-activated fibrinolysis inhibitor
THA: Total hip arthroplasty
TKA: Total knee arthroplasty
VTE: Venous thromboembolism
WBC: White blood cell
